# Supplementary material for: Effect of the PSSMA Content on the Heat Transfer Performances of Polyurea Nano-Encapsulated Phase Change Materials
Source: Materials (Basel). 2021 Jun 8;14(12):3157. doi: 10.3390/ma14123157 (PMC8227275; doi:10.3390/ma14123157)
Supplement: Supplementary file 1 [file materials-14-03157-s001.zip › materials-1215985-supplementary.pdf]

materials

Supplementary material

Effect of the PSSMA Content on the Heat Transfer PerformanceS of Polyurea Nano-encapsulated Phase Change Materials

Jun-Won Kook, Kiseob Hwang and Jun-Young Lee *

Research Institute of Sustainable Manufacturing Systems, Intelligent Sustainable materials R&D group, Korea Institute of Industrial Technology, 89 Yangdaegiro-gil, Ipjang-myeon, Seobuk-gu, Cheonan-si, Chungcheongnam-do 31056, Korea; kukjw83@kitech.re.kr (J.-W.K.); ks_hwang@kitech.re.kr (K.H.)
* Correspondence: jaylee@kitech.re.kr

Citation: Kook, J.-W.; Hwang, K.; Lee, J.-Y. Effect of the PSSMA Content on the Heat Transfer Performances of Polyurea Nano-encapsulated Phase Change Materials. *Materials* **2021**, *14*, 3157. https://doi.org/10.3390/ma14123157

Academic Editor: Maria Raimo

Received: 25 April 2021
Accepted: 5 June 2021
Published: 16 June 2021

Publisher's Note: MDPI stays neutral with regard to jurisdictional claims in published maps and institu-tional affiliations.

Copyright: © 2021 by the authors. Licensee MDPI, Basel, Switzerland. This article is an open access article distributed under the terms and conditions of the Creative Commons Attribution (CC BY) license (http://creativecommons.org/licenses/by/4.0/).

Thermal properties of the NEPCMs

To determine the thermal stabilities of the PCM and the shell material, the thermal properties of the prepared NEPCMs were evaluated by TGA; Figure A shows the TGA curves of the NEPCMs prepared by varying the PSSMA content. The thermal decomposition temperature of OD was ~150 °C, while PUA and PSSMA have thermal decomposition temperatures of 260–280 °C and 440–450 °C, respectively.

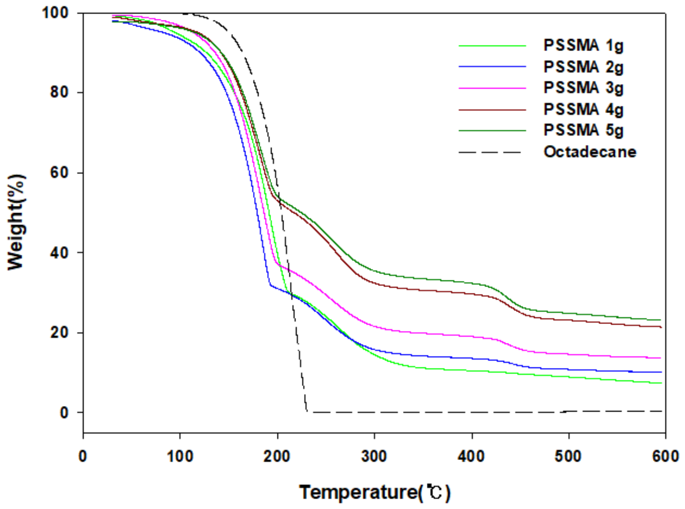

Figure A. TGA curves of the PUA-NEPCMs with varying PSSMA content.
